# Supplementary material for: Lipopolysaccharide Stimulates A549 Cell Migration through p-Tyr 42 RhoA and Phospholipase D1 Activity
Source: Biomolecules. 2023 Dec 20;14(1):6. doi: 10.3390/biom14010006 (PMC10813223; doi:10.3390/biom14010006)
Supplement: Supplementary file 1 [file biomolecules-14-00006-s001.zip › biomolecules-2766392-supplementary/biomolecules-2766392-supplementary.pdf]

Phosphatidic Acid (PA) binding proteins identification from A549 cell lysates

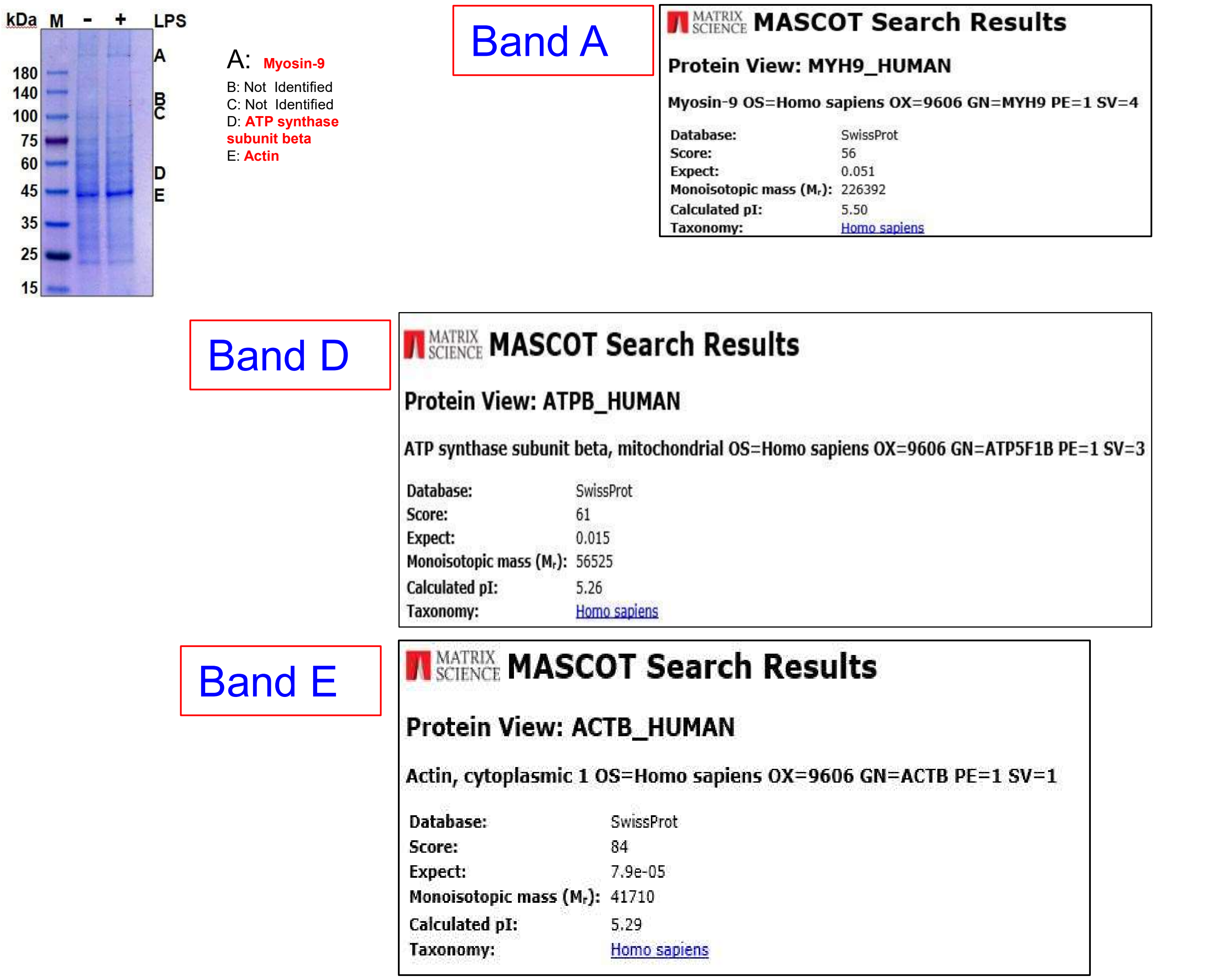

Supplementary Figure S1. Identification of proteins Binding to PA-conjugated beads. A549 cells were exposed to LPS (5 μg/ml) for 24 h to induce stimulation. Subsequently, the cell lysate was subjected to overnight incubation with phosphatidic acid (PA)-conjugated beads. The proteins were then separated using SDS-PAGE, and the identification of target protein species was performed through MALDI-TOF analysis. Bands A, D, and E were identified to be MYH9, ATP synthase subunit beta, and actin.

Interaction of PLD1 with PIP2, PIP3 and RhoA

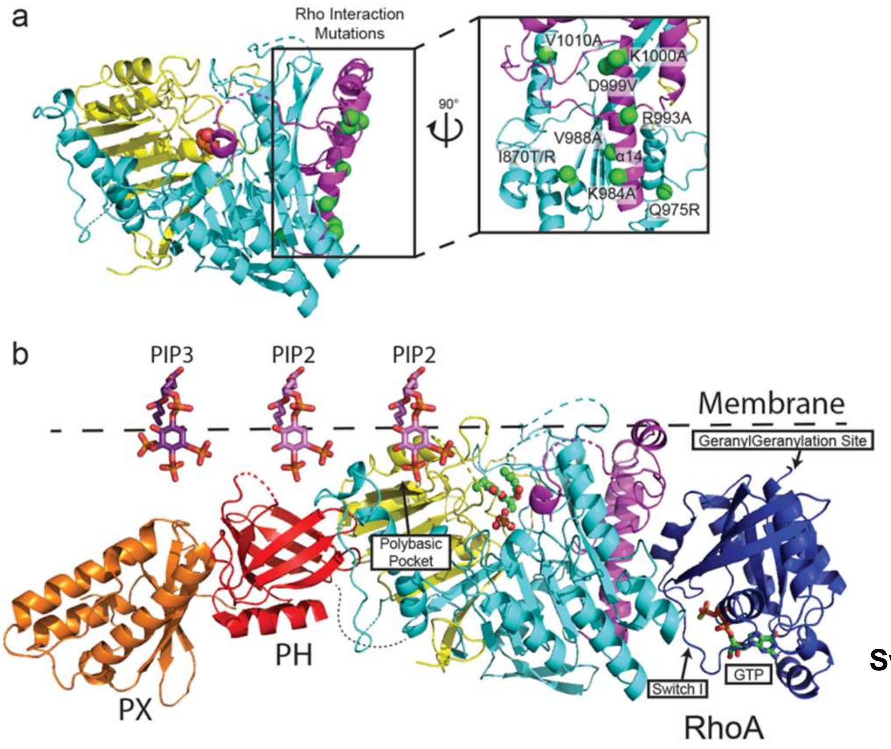

Figure 6. Model of RhoA activation of PLD1.

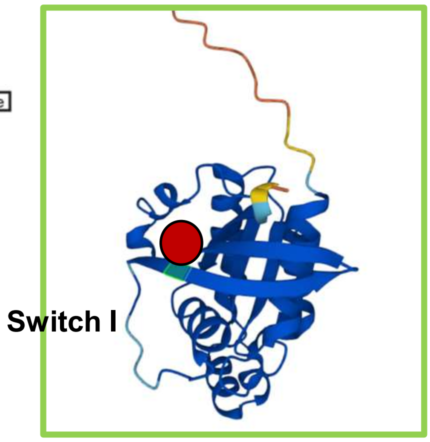

Nat Chem Biol. 2020. 16: 400–407.
doi:10.1038/s41589-020-0499-8.

[AlphaFold Protein Structure Database: RhoA]

P-Tyrosine 42 RhoA

Supplementary Figure S2. Three-dimensional structure of RhoA and PLD1 complex. The 3D structure of PLD1 and RhoA was presented in Nat Chem Biol. 2020. 16: 400–407. doi:10.1038/s41589-020-0499-8. We displayed the p-Tyr42 RhoA in the right panel. Red ball indicates p-Tyr42 residue of RhoA, which is located the end of switch I domain.
