# Supplementary figures and images for: Lipopolysaccharide Stimulates A549 Cell Migration through p-Tyr 42 RhoA and Phospholipase D1 Activity
Source: Biomolecules. 2023 Dec 20;14(1):6. doi: 10.3390/biom14010006 (PMC10813223; doi:10.3390/biom14010006)

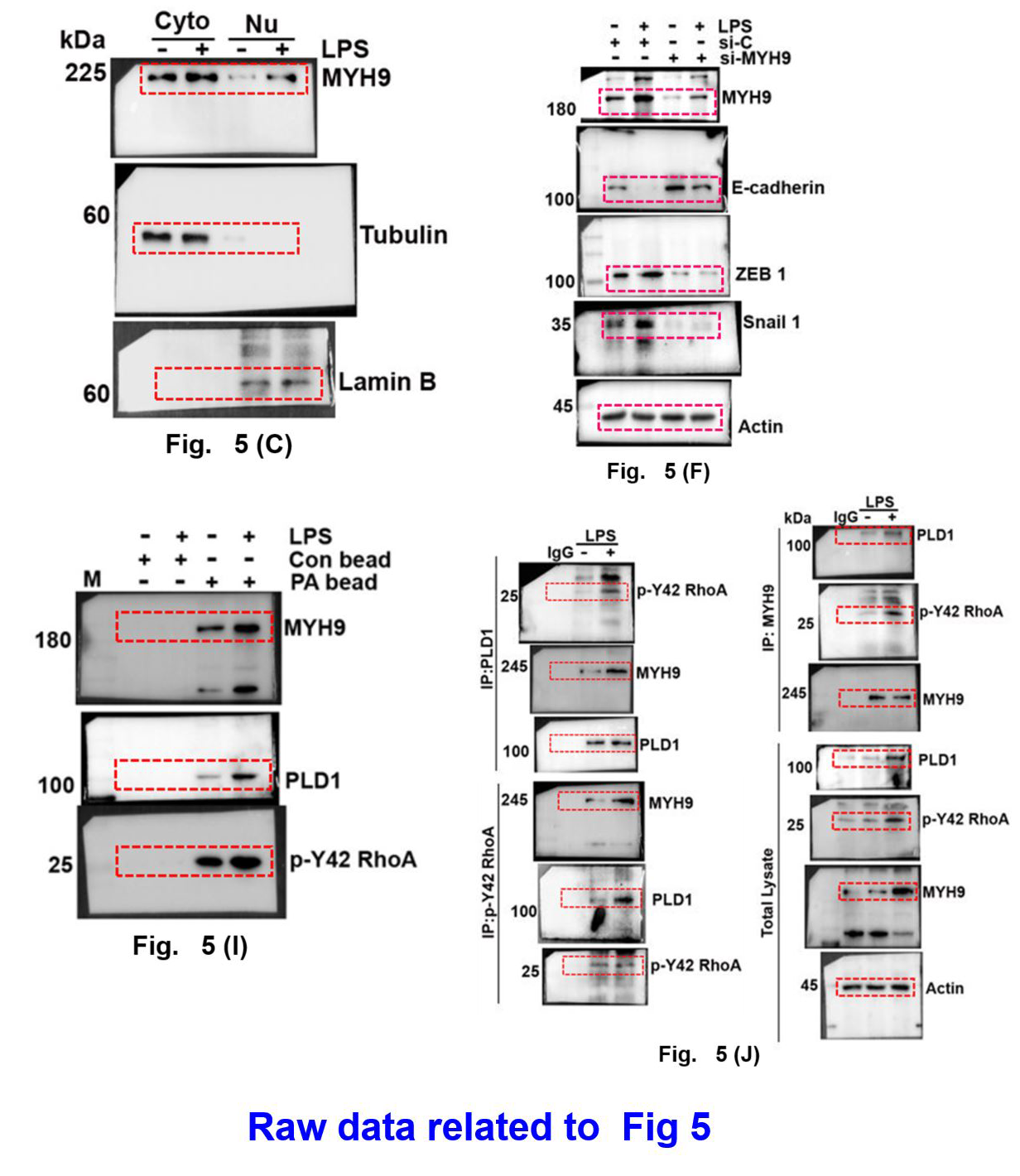

Supplement: Supplementary file 1 [file biomolecules-14-00006-s001.zip › biomolecules-2766392-supplementary/biomolecules-2766392-WB original/Figure S3 (3).tif]

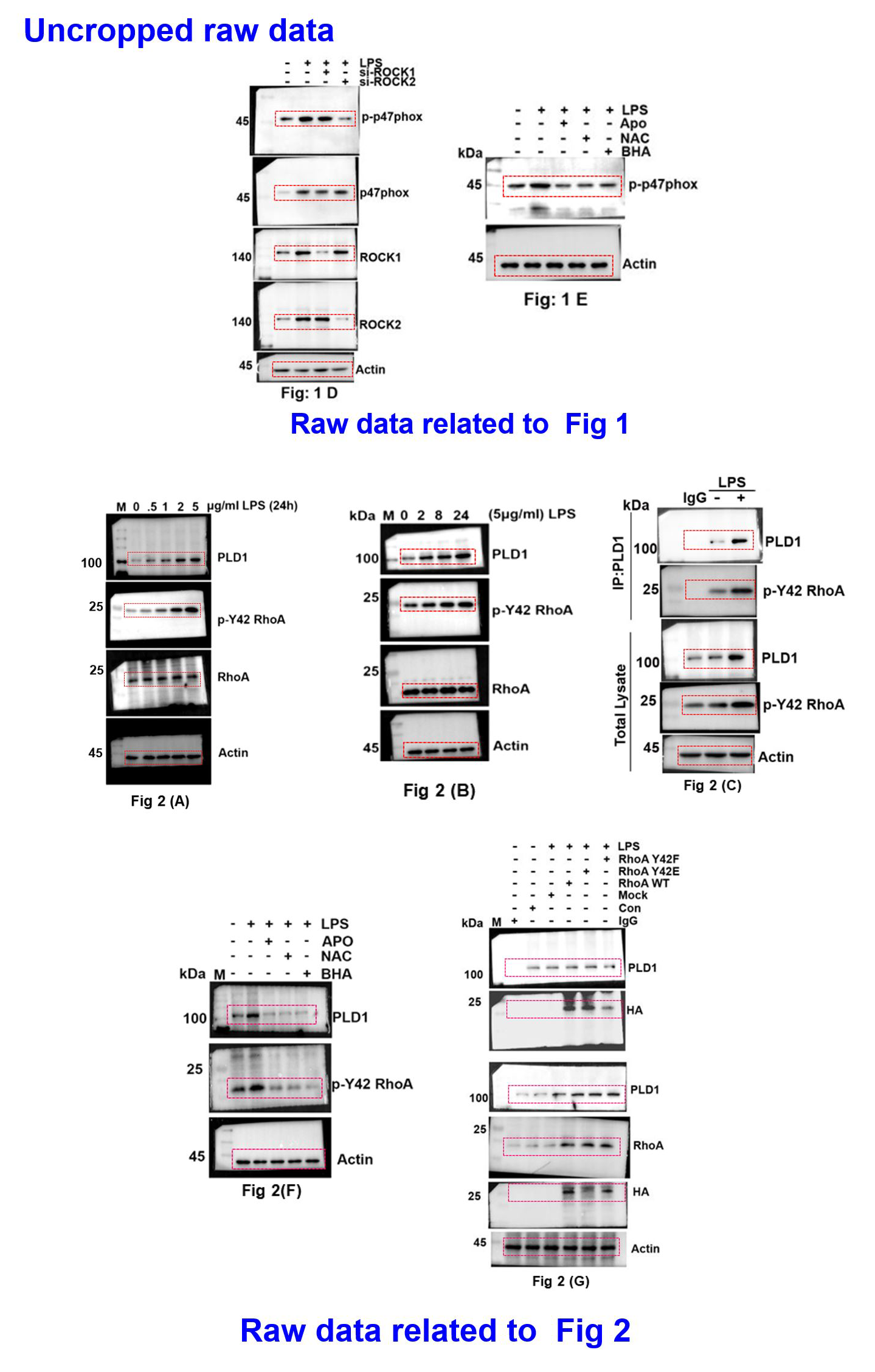

Supplement: Supplementary file 1 [file biomolecules-14-00006-s001.zip › biomolecules-2766392-supplementary/biomolecules-2766392-WB original/Figure S3(1).tif]

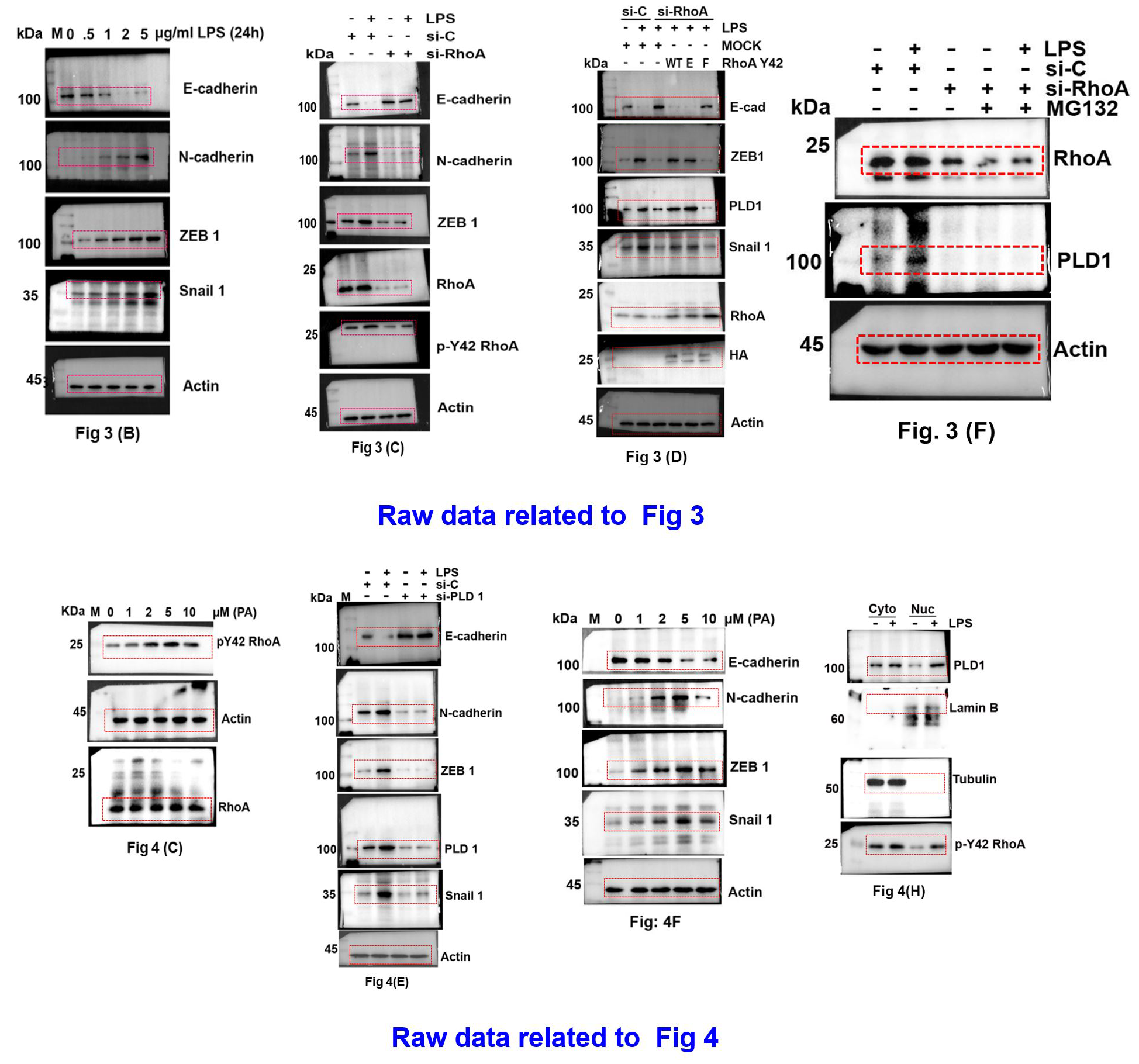

Supplement: Supplementary file 1 [file biomolecules-14-00006-s001.zip › biomolecules-2766392-supplementary/biomolecules-2766392-WB original/Figure S3(2) .tif]
